# Supplementary material for: Multi-pathway DNA-repair reporters reveal competition between end-joining, single-strand annealing and homologous recombination at Cas9-induced DNA double-strand breaks
Source: Nat Commun. 2022 Sep 8;13:5295. doi: 10.1038/s41467-022-32743-w (PMC9458747; doi:10.1038/s41467-022-32743-w)
Supplement: Supplementary file 1 — Supplementary Information [file 41467_2022_32743_MOESM1_ESM.pdf]

a

```
                10         20         30         40         50
eBFP1.2 MVSKGEELFT GVVPILVELD GDVNGHKFSV RGEGEGDATN GKLTLKFICT
eGFP    MVSKGEELFT GVVPILVELD GDVNGHKFSV SGEGEGDATY GKLTLKFICT

                60         70         80         90        100
eBFP1.2 TGKLPVPWPT LVTTLSHGVQ CFARYPDHMK QHDFFKSAMP EGYVQERTIF
eGFP    TGKLPVPWPT LVTTLTYGVQ CFSRYPDHMK QHDFFKSAMP EGYVQERTIF

               110        120        130        140        150
eBFP1.2 FKDDGTYKTR AEVKFEGDTL VNRIELKGID FKEDGNILGH KLEYNYNSHN
eGFP    FKDDGNYKTR AEVKFEGDTL VNRIELKGID FKEDGNILGH KLEYNYNSHN

               160        170        180        190        200
eBFP1.2 VYIMADKQKN GIKVNFKIRH NVEDGSVQLA DHYQQNTPIG DGPVLLPDSH
eGFP    VYIMADKQKN GIKVN----- ---------- ---------- ----------

               210        220        230
eBFP1.2 YLSTQSVLSK DPNEKRDHMV LLEFVTAAGI TLGMDELYK
eGFP    ---------- ---------- ---------- ---------
```

b

```
         L  V  T  T  L  S  H  G  V  Q  C  F  A  R  Y  P  D  H  M  K
eBFP1.2 CTCGTGACCACCCTCTCCCACGGGGTCCAGTGTTTTGCCCGGTATCCCGACCACATGAAG
eGFP    CTCGTGACCACCCTGACCTACGGCGTGCAGTGCTTCAGCCGCTACCCCGACCACATGAAG
```

Supplementary figure 1. Alignment of eBFP1.2 and eGFP sequences used in DSB-Spectrum. (a) Alignment of the amino acid sequence translated from the eBFP1.2 and eGFP genes present in the DSB-Spectrum_V2/V3 reporter constructs. The amino acids that differ between the two sequences are indicated in red. **(b)** Alignment of the DNA sequence of the eBFP1.2 and eGFP genes in the DSB-Spectrum_V2/V3 reporter constructs. Shown is the region that contains the BFP sgRNA target site. The sgRNA sequence is indicated by the blue box. The nucleotides that differ between the two sequences are indicated in red. Note that several silent mutations were introduced in the eGFP sequence to prevent any binding of the BFP-targeting sgRNA and cutting by Cas9.

a

Cell-line	Reporter	Location	Orientation	Genetic context
HEK 293T	DSB-Spectrum_V1	Chr20:35,500,035	-	*CEP250* gene, in intron
HEK 293T	DSB-Spectrum_V2	Chr14:61,715,736	-	*HIF1A* gene, in intron
HEK 293T	DSB-Spectrum_V3	Chr13:20,826,469	+	*XPO4* gene, in intron
U2OS	DSB-Spectrum_V2	Chr8:143,588,693	+	*EEF1D* gene, in intron

b

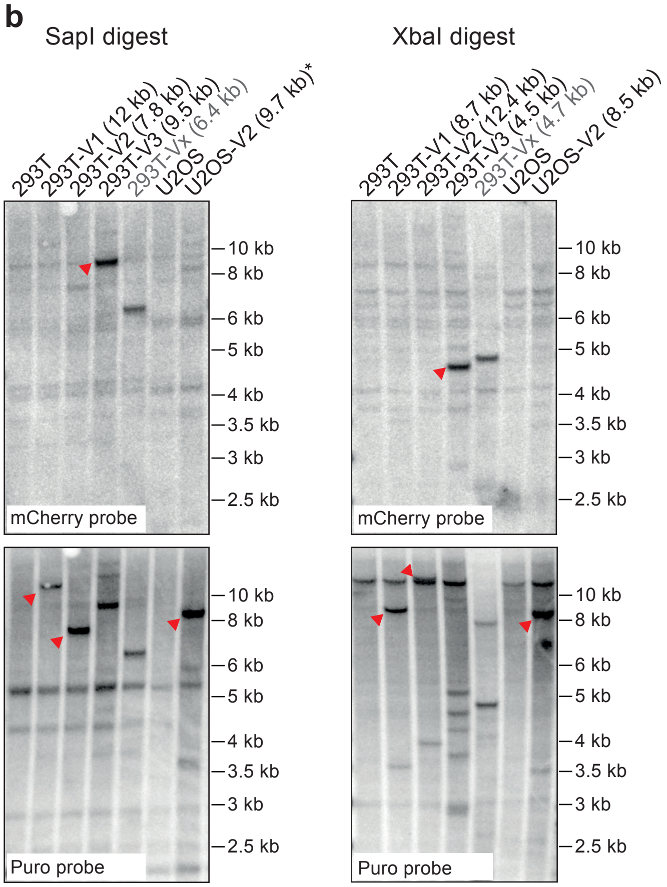

Supplementary figure 2. Identification and validation of DSB-Spectrum genomic integration sites (a) For the clonal DSB-Spectrum reporter cell-lines that were used in this manuscript, the genomic integration site of the reporter construct was determined by Splinkerette PCR as described in the methods section. **(b)** Genomic DNA isolated from clonal DSB-Spectrum reporter cell-lines, as well as the parental HEK 293T and U2OS cell-lines as a control, was digested overnight with either SapI or XbaI restriction enzyme, followed by southern blot analysis as indicated in the methods section. The blot was hybridized with a probe binding to the puromycin resistance cassette (puro probe) present in DSB-Spectrum variants V1 and V2, followed by stripping and re-hybridization with a probe binding to the mCherry gene (mCherry probe), present in DSB-Spectrum_V3. The sample loaded in lane 5 contains digested DNA from a HEK 293T cell-line containing a DSB-Spectrum variant that was discontinued and not described in the manuscript (293T-Vx). The expected size, based on the Splinkerette results, of the genomic DNA digestion product containing the DSB-Spectrum-derived puromycin resistance or mCherry gene is indicated in brackets behind the labels. Red arrows point towards the products specific for the DSB-Spectrum cell-lines compared to the parental control cell-lines. Asterisk indicates that the U2OS DSB-Spectrum_V2 cells were double digested with PacI in combination with XbaI. Data shown are representative for results obtained from five independently performed southern blots.

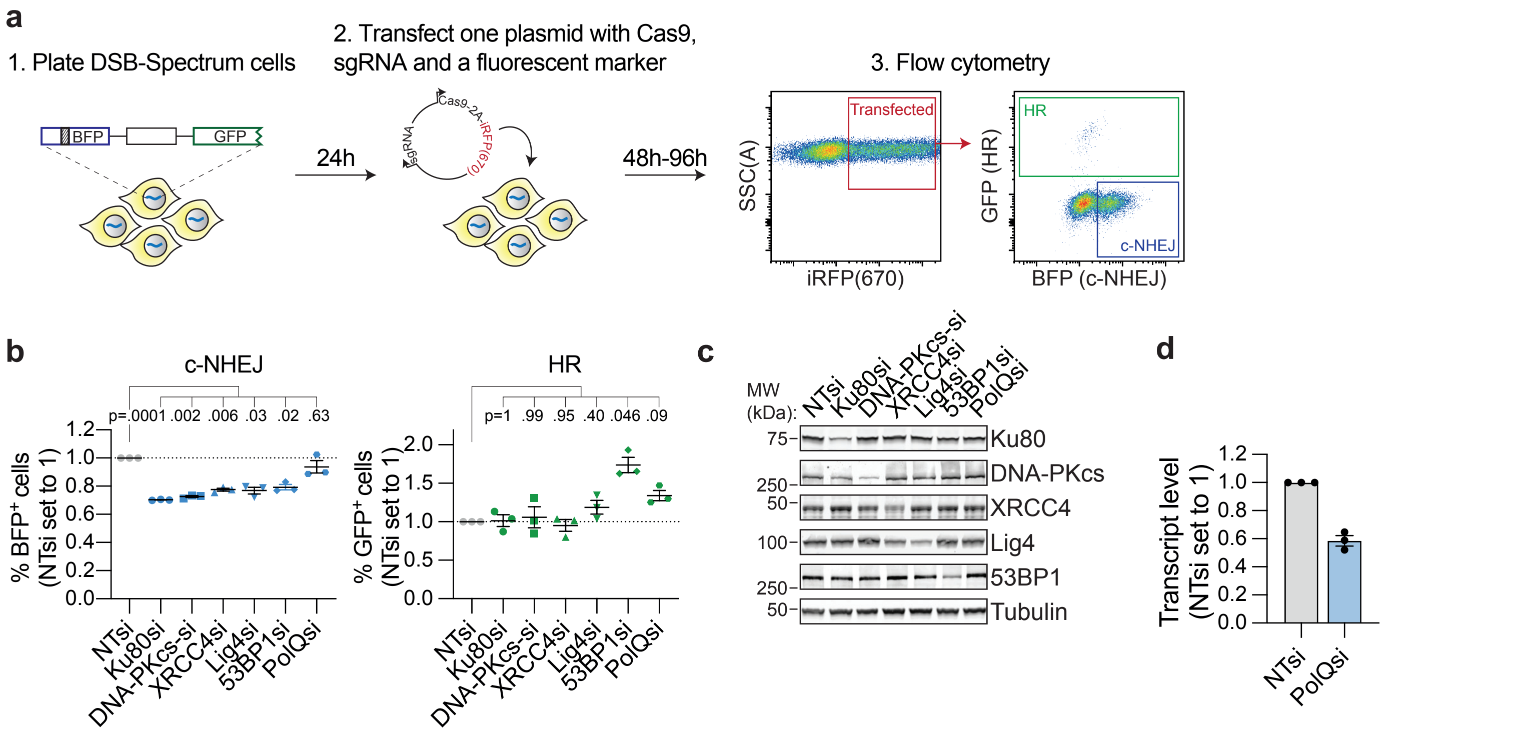

Supplementary figure 3. Validation of DSB-Spectrum_V1 (a) Lay-out of a typical experiment with DSB-Spectrum cells. **(b)** DSB-Spectrum_V1 cells were transfected with indicated siRNAs, followed by transfection with Cas9 and an sgRNA targeting either a control locus or BFP. At 72h after Cas9 transfection cells were analyzed by flow cytometry (n=3; mean ± SEM; One-way ANOVA, post-hoc Dunnett's). **(c)** Western blot of lysates from cells analyzed in panel b. **(d)** Quantification of PolQ (PolΘ) knock-down in cells used in panel b. RNA was isolated, followed by reverse transcription and quantitative PCR. Plotted are the PolQ transcript levels normalized to GAPDH transcript levels (n=3; mean ± SEM). Source data for panels b and d are provided as a Source Data file.

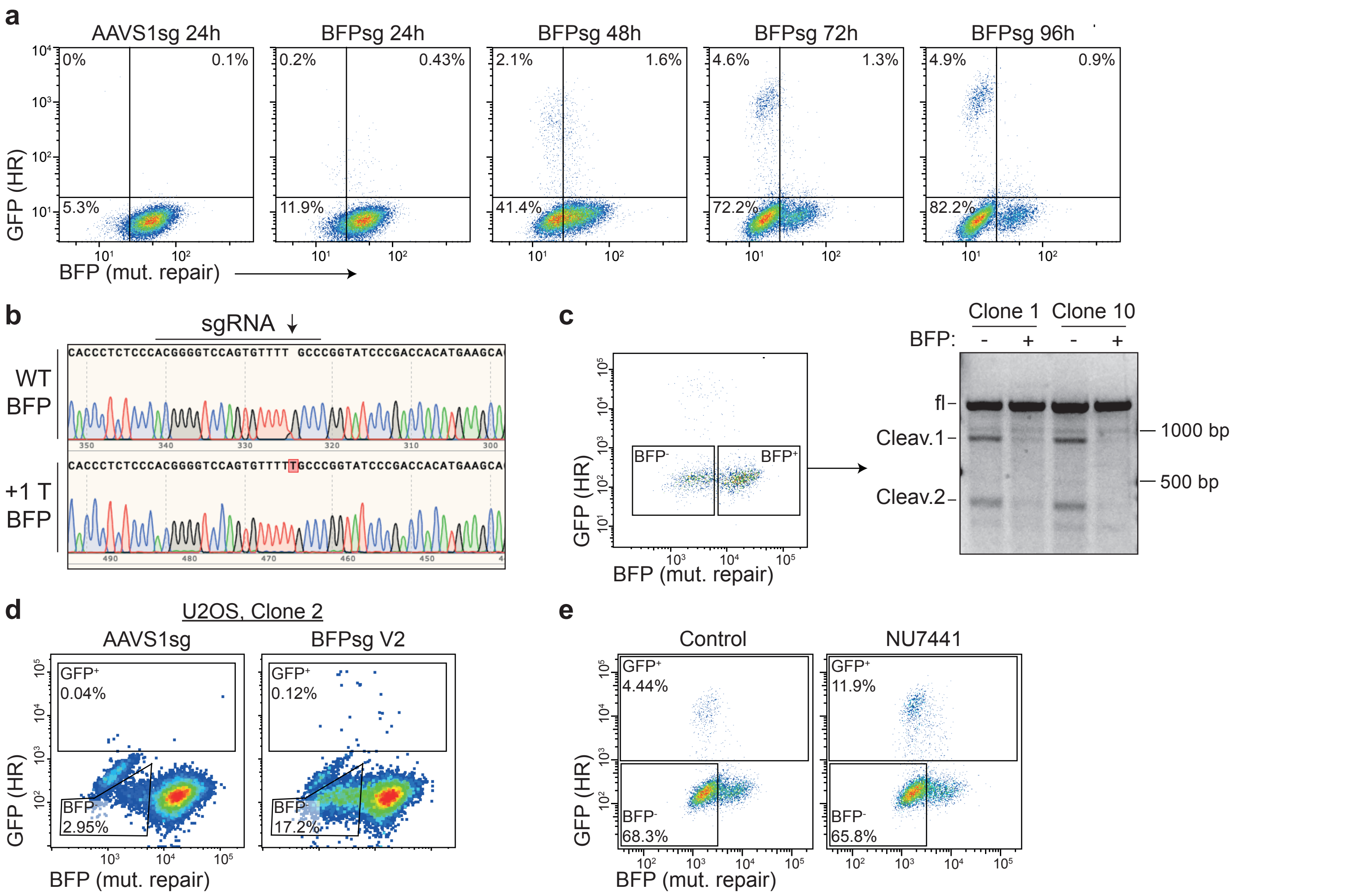

Supplementary figure 4. Validation of DSB-Spectrum_V2 (a) DSB-Spectrum_V2 cells were transfected with Cas9 and an sgRNA targeting either a control locus or BFP. At indicated time points after Cas9 transfection cells were analyzed by flow cytometry. **(b and c)** HEK 293T DSB-Spectrum_V2 cells were transfected with Cas9 and a BFP-targeting sgRNA, followed by FACS to collect the BFP^- and BFP^+ populations, and subsequent PCR amplification of the Cas9 target locus. The PCR product of the BFP^- population was TOPO-cloned followed by Sanger-sequencing of plasmid DNA preps from individual bacterial colonies. Panel b displays a screenshot of the aligned sequencing results of a WT and +1T insertion repair product, which was the most frequent InDel identified in the BFP^- population. In addition, the PCR products from both the BFP^- and BFP^+ populations were denatured and re-annealed, followed by Surveyor nuclease digestion and electrophoretic analysis of the digestion products, shown in panel c. Clone 1 and clone 10 refers to two different clonally derived DSB-Spectrum_V2 cell-lines. **(d)** A second U2OS DSB-Spectrum_V2 cell-line, derived from a different clone than the U2OS DSB-Spectrum_V2 cell-line shown in figure 2, was transfected with Cas9 and either a control sgRNA (AAVS1sg) or a BFP-targeting sgRNA, followed by flow cytometric analysis 72h later. Shown is a representative flow cytometry plot. **(e)** Representative flow cytometry plots of the experiments shown in figure 2i.

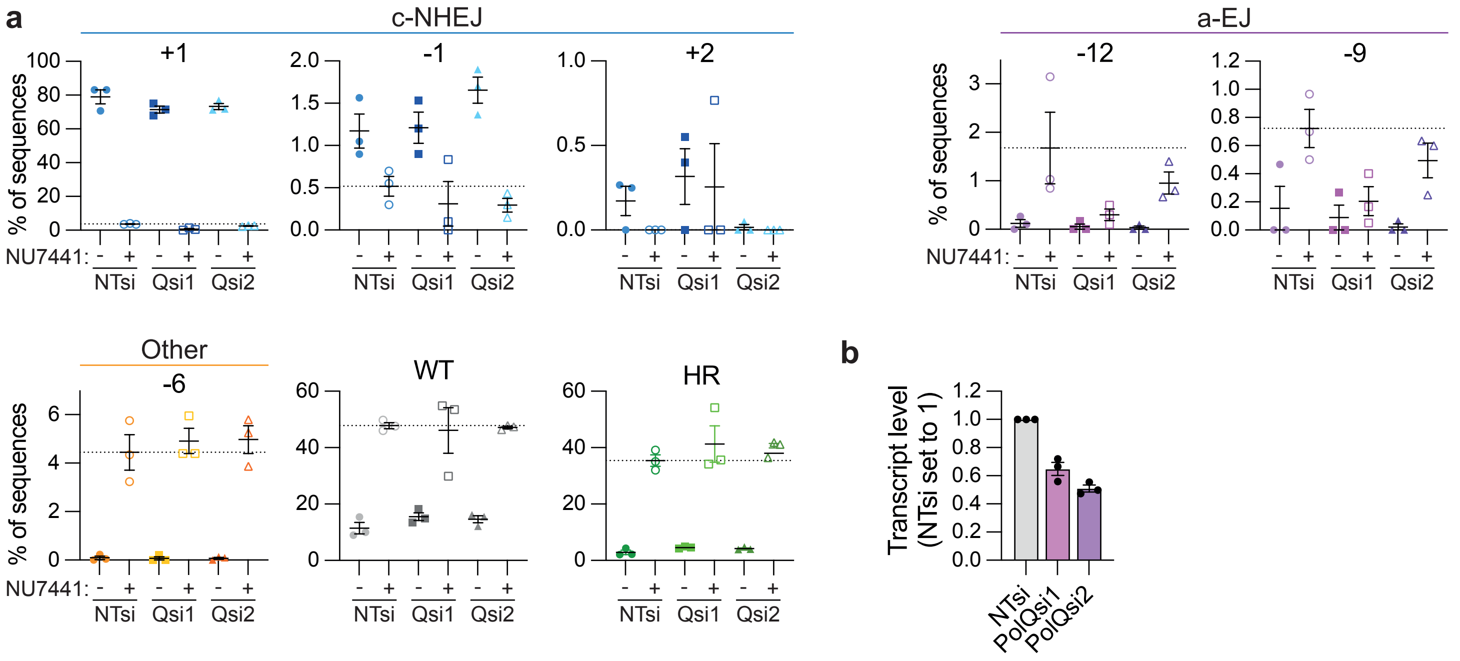

Supplementary figure 5. The contribution of a-EJ to DSB-repair of DSB-Spectrum_V2 is minor. (a) The data depicted in figure 3a were replotted to show all frequencies for every individual sequence detected by TIDER anaysis. NTsi=Non-Targeting control siRNA, Qsi=PolQ-targeting siRNA. (n=3, mean ± SEM). **(b)** RNA was extracted from the cells described in figure 3a, followed by qRT-PCR to determine PolQ transcript levels (n=3, mean ± SEM). Source data for all panels are provided as a Source Data file.

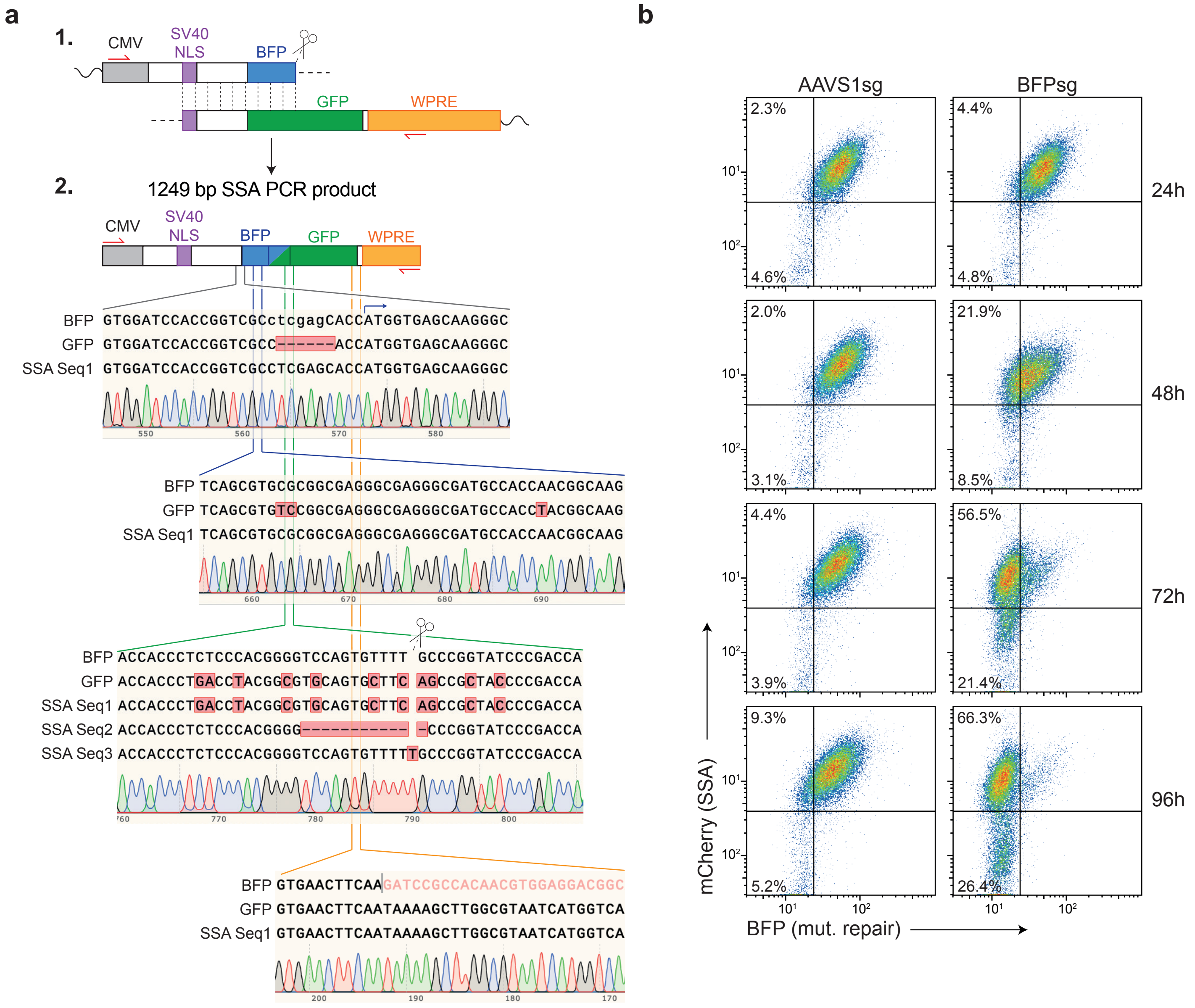

Supplementary figure 6. Repair of DSB-Spectrum by SSA is frequent. (a) Diagram 1 shows a schematic of the homologous regions that are predicted to anneal during SSA-repair of DSB-Spectrum_V2. The red primers were used to PCR-amplify the 1249 bp repair product described in figure 3d, followed by sequencing. The result is shown in diagram 2, including sequence alignments between the BFP and GFP regions in DSB-Spectrum, and sequences of the SSA repair product. Four alignments of different areas are shown, which together indicate that the SSA product perfecty aligns to the BFP and GFP gene regions at the 5'-end and the 3'-end of the DSB-site, repectively. Note that sequence variation between individual SSA DSB-repair products exists at the region surrounding the Cas9 target site. Sequencing chromatograms are of the SSA repair product. **(b)** DSB-Spectrum_V3 cells were transfected with Cas9 and an sgRNA targeting either a control locus or BFP. At indicated time points after Cas9 transfection cells were analyzed by flow cytometry.

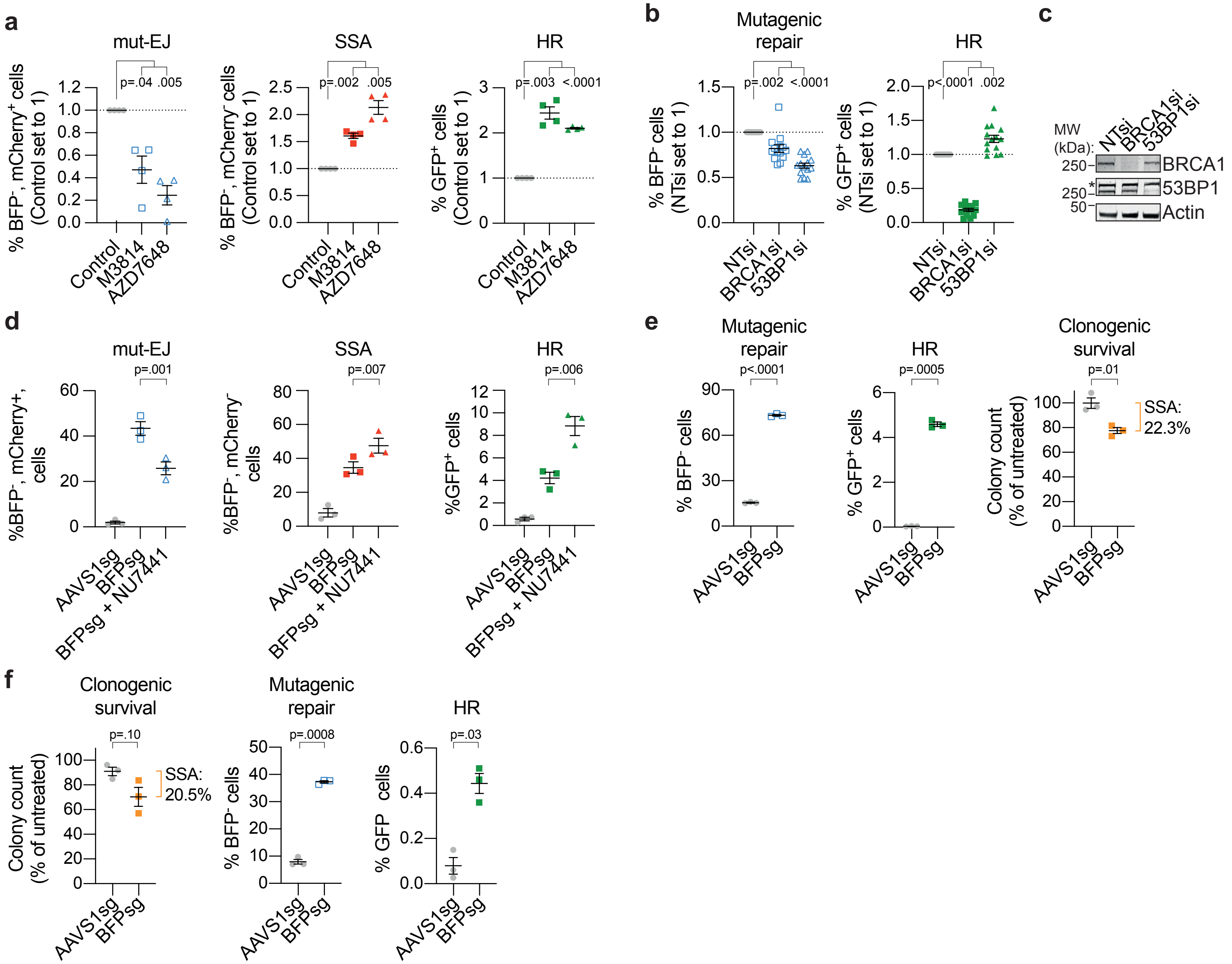

Supplementary figure 7. Inhibition of DNA-PKcs increases SSA-repair of DSB-Spectrum. (a) HEK 293T DSB-Spectrum_V3 cells were transfected with Cas9 and an sgRNA targeting either AAVS1 or BFP, followed by treatment with the DNA-PKcs inhibitors M3841 (2 µM) or AZD7648 (2 µM). At 72h after Cas9 transfection cells were analyzed by flow cytometry (n=4; mean ± SEM; One-way ANOVA, post-hoc Dunnett's). **(b)** BRCA1 and 53BP1 expression was silenced by RNAi, and mutagenic repair and HR were quantified as in figure 1e but for DSB-Spectrum_V2 cells (n=14; mean ± SEM; One-way ANOVA, post-hoc Dunnett's). **(c)** Western blot of lysates from cells analyzed in panel B. Asterisk indicates non-specific background band. **(d)** Experiment performed as in figure 4d, in a separate HEK 293T DSB-Spectrum_V3 clone (n=3; mean ± SEM; paired t-test, two-tailed). **(e)** HEK 293T DSB-Spectrum_V2 cells were transfected with Cas9 cDNA and an sgRNA targeting either a control locus (AAVS1sg) or DSB-Spectrum_V2 (BFPsg). At 72h post-transfection, cells were harvested and either analyzed by flow cytometry to quantify mutagenic repair and HR, or replated for a clonogenic survival assay in presence or absence of puromycin, to measure SSA based on loss of the puromycin resistance gene. The number of colonies in the puromycin-treated plates were normalized to the number of colonies observed in control plates lacking puromycin (n=3; mean ± SEM; paired t-test, two-tailed). **(f)** As in panel e, but for U2OS DSB-Spectrum_V2 cells (n=3; mean ± SEM; paired t-test, two-tailed). Source data for panels a, b, d, e and f are provided as a Source Data file.

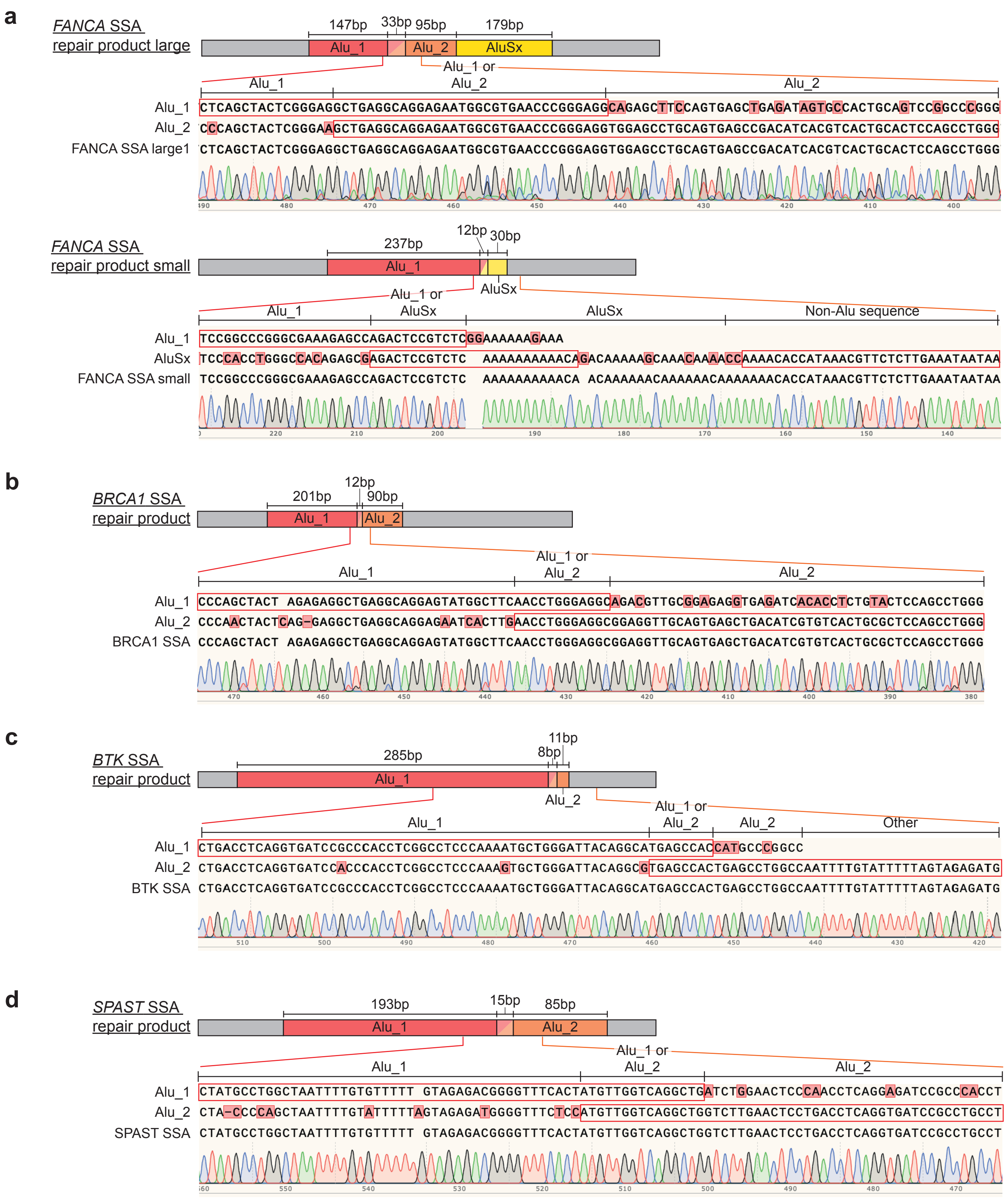

Supplementary figure 8. Sequence validation of the *FANCA, BRCA1, BTK* and *SPAST* Alu SSA DSB-repair products. (a-d) The *FANCA, BRCA1, BTK* and *SPAST* Alu SSA repair products that were PCR amplified as described in figure 5 were TOPO-cloned and analyzed by Sanger sequencing. The composition of the SSA repair products is displayed as cartoons, shown below these cartoons is a sequence alignment of the individual Alu elements to the sequence of the SSA repair product, focussing on the junction region between the two recombined Alu elements. Regions in each Alu element that align perfectly to the SSA repair product are indicated by the red box. Mismatches between the Alu_1/Alu_2 sequence and the SSA repair product sequence are indicated by red shading. Below the alignment a representative sequence chromatogram of the SSA repair product is shown. Note that Alu sequences at the junction that share 100% homology can be derived from either Alu_1 or Alu_2.
