# Supplementary material for: Multi-pathway DNA-repair reporters reveal competition between end-joining, single-strand annealing and homologous recombination at Cas9-induced DNA double-strand breaks
Source: Nat Commun. 2022 Sep 8;13:5295. doi: 10.1038/s41467-022-32743-w (PMC9458747; doi:10.1038/s41467-022-32743-w)
Supplement: Supplementary file 3 — Reporting Summary [file 41467_2022_32743_MOESM3_ESM.pdf]

nature portfolio

Corresponding author(s): Michael B. Yaffe

Last updated by author(s): Jul 28, 2022

Reporting Summary

Nature Portfolio wishes to improve the reproducibility of the work that we publish. This form provides structure for consistency and transparency in reporting. For further information on Nature Portfolio policies, see our Editorial Policies and the Editorial Policy Checklist.

Statistics

For all statistical analyses, confirm that the following items are present in the figure legend, table legend, main text, or Methods section.

n/a	Confirmed	
☐	☒	The exact sample size (*n*) for each experimental group/condition, given as a discrete number and unit of measurement
☐	☒	A statement on whether measurements were taken from distinct samples or whether the same sample was measured repeatedly
☐	☒	The statistical test(s) used AND whether they are one- or two-sided<br>*Only common tests should be described solely by name; describe more complex techniques in the Methods section.*
☐	☒	A description of all covariates tested
☐	☒	A description of any assumptions or corrections, such as tests of normality and adjustment for multiple comparisons
☐	☒	A full description of the statistical parameters including central tendency (e.g. means) or other basic estimates (e.g. regression coefficient) AND variation (e.g. standard deviation) or associated estimates of uncertainty (e.g. confidence intervals)
☐	☒	For null hypothesis testing, the test statistic (e.g. *F*, *t*, *r*) with confidence intervals, effect sizes, degrees of freedom and *P* value noted<br>*Give P values as exact values whenever suitable.*
☒	☐	For Bayesian analysis, information on the choice of priors and Markov chain Monte Carlo settings
☒	☐	For hierarchical and complex designs, identification of the appropriate level for tests and full reporting of outcomes
☒	☐	Estimates of effect sizes (e.g. Cohen's *d*, Pearson's *r*), indicating how they were calculated

Our web collection on statistics for biologists contains articles on many of the points above.

Software and code

Policy information about availability of computer code

Data collection	1. FACS DIVA Software 5.0.3 (flow cytometry)<br>2. LI-COR ImageStudio ver. 5.2 (western blot)
Data analysis	1. FlowJo 10.8.0 (flow cytometry)<br>2. LI-COR ImageStudio Lite 5.2.5 (western blot)<br>3. BioRad CFX Manager Software ver. 3.1 (qPCR)<br>4. GraphPad Prism 9.3.0 (plotting and statistics)<br>5. SnapGene 6.0.5 (DNA cloning design and sequence alignment)<br>6. TIDER: Tracking of Insertion, DEletions and Recombination events, https://tide.nki.nl

For manuscripts utilizing custom algorithms or software that are central to the research but not yet described in published literature, software must be made available to editors and reviewers. We strongly encourage code deposition in a community repository (e.g. GitHub). See the Nature Portfolio guidelines for submitting code & software for further information.

Data

Policy information about availability of data

All manuscripts must include a data availability statement. This statement should provide the following information, where applicable:

- Accession codes, unique identifiers, or web links for publicly available datasets
- A description of any restrictions on data availability
- For clinical datasets or third party data, please ensure that the statement adheres to our policy

All source data are provided with this paper.

Human research participants

Policy information about studies involving human research participants and Sex and Gender in Research.

Reporting on sex and gender	N/A
Population characteristics	N/A
Recruitment	N/A
Ethics oversight	N/A

Note that full information on the approval of the study protocol must also be provided in the manuscript.

Field-specific reporting

Please select the one below that is the best fit for your research. If you are not sure, read the appropriate sections before making your selection.

☒ Life sciences ☐ Behavioural & social sciences ☐ Ecological, evolutionary & environmental sciences

For a reference copy of the document with all sections, see nature.com/documents/nr-reporting-summary-flat.pdf

Life sciences study design

All studies must disclose on these points even when the disclosure is negative.

Sample size	No statistical method was used to predetermine sample size. Consistent with common practice in molecular biology, a minimum sample size of n=3 was chosen for all experiments that required quantitative and statistical analysis. This includes DSB-Spectrum reporter assays, TIDER analysis and gel-based quantification of SSA PCR products. For some experiments with relatively large data spread, the sample size was increased to more reliably determine the mean and statistics. For a few experiments that required only qualitative data interpretation, or less stringent quantitative data interpretation, including western blot and qRT-PCR analysis of knockdown efficiency, the sample size was smaller than n=3.
Data exclusions	No data were excluded
Replication	All experiments for which statistical analysis is included were successfully replicated for at least three experiments. All other experiments were successfully replicated at least twice.
Randomization	There was no allocation of test subjects for any of the experiments, thus randomization was not applicable to our study. We work with cell cultures and compare specific experimental conditions or wild type versus knockdown/knockout cell lines, which cannot be randomized.
Blinding	Experiments were performed and analyzed by the same person, and were small scale including less then 10 conditions. Thus, careful labeling of samples throughout the experiment and analysis prohibited blinding. To nonetheless ensure unbiased analysis, all quantification was done with analysis software (FlowJo, ImageStudio, etc.) using defined parameters that were identical between all conditions within an experiment.

Reporting for specific materials, systems and methods

We require information from authors about some types of materials, experimental systems and methods used in many studies. Here, indicate whether each material, system or method listed is relevant to your study. If you are not sure if a list item applies to your research, read the appropriate section before selecting a response.

Materials & experimental systems

n/a	Involved in the study
☐	☒ Antibodies
☐	☒ Eukaryotic cell lines
☒	☐ Palaeontology and archaeology
☒	☐ Animals and other organisms
☒	☐ Clinical data
☒	☐ Dual use research of concern

Methods

n/a	Involved in the study
☒	☐ ChIP-seq
☐	☒ Flow cytometry
☒	☐ MRI-based neuroimaging

Antibodies

Antibodies used

1. anti-BRCA1 (Rabbit polyclonal, Millipore 07-434)
2. anti-53BP1 (Rabbit polyclonal, Novus NB100-304)
3. anti-Ku80 (rabbit polyclonal, Santa Cruz sc-9034)
4. anti-DNA-PKcs (Mouse monoclonal, Abcam ab44815, clone 18-2)
5. anti-XRCC4 (mouse monoclonal, SAB Signalway 40455 (no clone information provided by manufacturer))
6. anti-LIG4 (rabbit monoclonal, Abcam ab193353, clone EPR16531)
7. anti-alpha-Tubulin (mouse monoclonal, Sigma, clone DM1A, T6199)
8. anti-β-Actin (Mouse monoclonal, Millipore Sigma Cat No. A5441, clone AC-15)
9. anti-BRCA2 (Mouse monoclonal, Merck-Millipore OP95, clone 2B)
10. anti-Rad52 (Mouse monoclonal, Santa-Cruz sc-365341, clone F-7)
11. anti-Exo1 (Rabbit polyclonal, Abcam ab95068)
12. anti-CtIP (Mouse monoclonal, Millipore MABE1060, clone 14-1)
13. anti-Mre11 (Kind gift from prof. Roland Kanaar, Erasmus MC, Rotterdam, the Netherlands; described in: de Jager, M. et al. DNA-binding and strand-annealing activities of human Mre11: implications for its roles in DNA double-strand break repair pathways. Nucleic Acids Research 29, 1317–1325 (2001))
14. Goat-anti-Rabbit IRDye 800CW (Goat polyclonal, LI-COR 926-22211)
15. Goat-anti-Mouse IRDye 680RD (Goat polyclonal, LI-COR 926-68070)

Validation

All antibodies were validated by us using siRNA-mediated knockdown, with the exception of the anti-tubulin and anti-β-Actin antibodies (listed as #7 and #8 above).

1. anti-BRCA1. The manufacturer indicates that the antibody works in WB of human samples. It provides a list of 21 references on its website that used the antibody. On the manufacturer's website, a western blot image is shown indicating BRCA1 detection in HeLa nuclear extract. In the manuscript, we validate it by siRNA-mediated knockdown.

2. anti-53BP1: There are two user reviews on the manufacturer's website that rate the antibody at least 4 out of 5 stars for use in western blot. The manufacturer provides a list of 79 references on its website that cite human and mouse reactivity in WB of the antibody. On the manufacturer's website, there are multiple western blot images derived from publications that show 53BP1 detection in human cell lysates by this antibody. In the manuscript, we validate it by siRNA-mediated knockdown.

3. anti-Ku80: The manufacturer mentions 27 citations of this product. The overall rating of the antibody performance is 5 out of 5 (10 user reviews). In the manuscript, we validate it by siRNA-mediated knockdown.

4. anti-DNA-PKcs: The manufacturer indicates that the antibody is KO validated. A user review on the manufacturer's website rates it 5 out of 5 stars for western blot application. A western blot image is included on the manufacturer's website showing detection of DNA-PKcs on western blot of HAP1 lysates, including loss of the detected band in DNA-PKcs KO cells. In the manuscript, we validate it by siRNA-mediated knockdown.

5. anti-XRCC4: The manufacturer's website includes western blot images showing antibody performance on human and mouse tissue lysates. In the manuscript, we validate it by siRNA-mediated knockdown.

6. anti-LIG4: The manufacturer indicates the antibody is KO validated, and shows a western blot image with the evidence of this validation on the website. It provides a list of 11 publications that have used the antibody. In the manuscript, we validate it by siRNA-mediated knockdown.

7. anti-alpha-Tubulin: The manufacturer indicates this is an Enhanced validation antibody. On it's website, the manufacturer provides multiple western blot imaging as evidence for succesfull western blot application of the antibody.

8. anti-β-Actin: The manufacturer's website includes a western blot image indicating good antibody performance, detecting Actin in cell lysates from nine different cell-lines. The antibody was cited by PMID: 19756912.

9. anti-BRCA2: Cited in Andres, J.L., et al. 1998. Oncogene 16, 2229.; Chen, J., et al. 1998. Mol. Cell 2, 317.; Chen, P.L., et al. 1998. Proc. Natl. Acad. Sci. USA 95, 5287. ;Marmorstein, L.Y., et al. 1998. Proc. Natl. Acad. Sci. USA 95, 13869. ;Connor, F., et al. 1997. Nat. Genet. 17, 423.; Sharan, S.K., et al. 1997. Nature 386, 804.; Ludwig, T., et al. 1997. Genes Dev. 11, 1226.; Mizuta, R., et al. 1997. Proc. Natl. Acad. Sci. USA 94, 6927.; Rajan, J.V., et al. 1997. Dev. Biol. 184, 385.; Suzuki, A., et al. 1997. Genes Dev. 11, 1242.; Wong, A.K.C., et al. 1997. J. Biol. Chem. 272, 31941.; Cannon-Albright, L.A. and M.H. Skolnick. 1996. Semin. Oncol. 23, 1.; Goggins, M., et al. 1996. Cancer Res. 56, 5360.; Stratton, M.R. 1996. Hum. Mol. Genet. 5, 1515.; Tavtigian, S.V., et al. 1996. Nat. Genet. 12, 333.; Rajan, J.V., et al. 1996. Proc. Natl. Acad. Sci. USA 93, 13078.; Spillman, M.A. and A.M. Bowcock. 1996. Oncogene 13, 1639.; Vaughn, J.P., et al. 1996.

Cancer Res. 56, 4590.; Wooster, R., et al. 1995. Nature 378, 789. In the manuscript, we validate it by siRNA-mediated knockdown.

10. anti-Rad52: The manufacturer's website provides a western blot image indicating good performance of this antibody, detecting Rad52 in five different human cell-line lysates. The antibody was cited 39 times, according to the manufacturer, and was rated 4.4 out of 5 by users. In the manuscript, we validate it by siRNA-mediated knockdown.

11. anti-Exo1: The manufacturer's website includes a western blot image validating anti-body detection of Exo1 in HeLa and 293T whole cell lysates. One user reviews the antibody performance 5 out of 5 stars, and the manufacturer indicates that 6 publications cite this antibody. In the manuscript, we validate it by siRNA-mediated knockdown.

12. anti-CtIP: The manufacturer cites the following publications that succesfully used the antibody for CtIP detection in western blot applications (Yu, X., et. al. (2000). J Biol Chem. 275(24):18541-9; Bunting, S.F., et. al. (2010). Cell. 141(2):243-54; Wu, M., et. al. (2007). Mol Cancer Res. 5(12):1285-95).

13. anti-Mre11: This antibody was validated in the original publication (de Jager, M. et al. DNA-binding and strand-annealing activities of human Mre11: implications for its roles in DNA double-strand break repair pathways. Nucleic Acids Research 29, 1317–1325 (2001). In the manuscript, we validate it by siRNA-mediated knockdown.

14. and 15. Goat-anti-Rabbit/Mouse secondary antibodies. The manufacturer states the following information on its website regarding validation of the antibody: Based on ELISA and flow cytometry, this antibody reacts with the heavy and light chains of rabbit IgG, and with the light chains of rabbit IgM and IgA. This antibody was tested by dot blot and and/or solid-phase adsorbed for minimal cross-reactivity with human, mouse, rat, sheep, and chicken serum proteins, but may cross-react with immunoglobulins from other species. The conjugate has been specifically tested and qualified for Western blot and In-Cell Western™ assay applications.

Eukaryotic cell lines

Policy information about cell lines and Sex and Gender in Research

Cell line source(s)	U-2 OS (female) and HEK 293T (female) cells were originally purchased from ATCC (codes HTB-96 and CRL-3216, respectively). Acquisition date and passage number since purchase was not indicated.
Authentication	Cell-lines were not authenticated
Mycoplasma contamination	All cell-lines were routinely tested for mycoplasm infection and have always tested negative.
Commonly misidentified lines (See ICLAC register)	No commonly misidentified cell-lines were used in this study.

Flow Cytometry

Plots

Confirm that:

☒ The axis labels state the marker and fluorochrome used (e.g. CD4-FITC).

☒ The axis scales are clearly visible. Include numbers along axes only for bottom left plot of group (a 'group' is an analysis of identical markers).

☒ All plots are contour plots with outliers or pseudocolor plots.

☒ A numerical value for number of cells or percentage (with statistics) is provided.

Methodology

Sample preparation	HEK 293T or U-2 OS cells were cultured as a monolayer and harvested by trypsinization. Next, trypsin was quenched and diluted by addition of a 4x volume PBS+1% BSA, resuspended and kept on ice. Immediately before sample acquisition, cells were resuspended and filtered using a 35 micrometer strainer cap tubes.
Instrument	BD LSRFortessa™ X-20 Cell Analyzer
Software	The Fortessa was running FACS Diva software, continuously updated. Data was analyzed using FlowJo 10.8.0
Cell population abundance	Cells were harvested from 96-well plates (HEK 293T) or 12-well plates (U-2 OS). Between 5,000 and 20,000 target events were acquired.
Gating strategy	1. Gating on FCS-A vs SSC-A to gate on live cells. 2. Gating on SSC-H vs SSC-W to exclude doublets. 3. Gating on FSC-H vs FSC-W to exclude doublets. 4. Gating on iRFP(670) positive (640 nm laser, APC filter settings) to select for iRFP(670) cells. 4. Gating on mut-EJ, SSA or HR populations as detailed in the manuscript.

☒ Tick this box to confirm that a figure exemplifying the gating strategy is provided in the Supplementary Information.
